# Intraoperative Hemorrhage and Postoperative Sequelae after Intraoral Vertical Ramus Osteotomy to Treat Mandibular Prognathism

**DOI:** 10.1155/2015/318270

**Published:** 2015-10-12

**Authors:** Chun-Ming Chen, Steven Lai, Ker-Kong Chen, Huey-Er Lee

**Affiliations:** ^1^Department of Oral and Maxillofacial Surgery, Kaohsiung Medical University Hospital, Kaohsiung Medical University, Taiwan; ^2^Department of Conservative Dentistry, Kaohsiung Medical University Hospital, Kaohsiung Medical University, Taiwan; ^3^Graduate Institute of Dental Sciences, College of Dental Medicine, Kaohsiung Medical University, Taiwan

## Abstract

*Objective.* To investigate the factors affecting intraoperative hemorrhage and postoperative sequelae after orthognathic surgery. *Materials and Methods.* Eighty patients with mandibular prognathism underwent surgical mandibular setback with intraoral vertical ramus osteotomy (IVRO). The correlation between the blood loss volume and postoperative VAS with the gender, age, and operating time was assessed using the *t*-test and Spearman rank correlation coefficient. The correlation between the magnitude of mandibular setback with the presence of TMJ clicking symptoms and lip sensation was also assessed. *Results.* The mean operating time and blood loss volume for men and women were 249.52 min and 229.39 min, and 104.03 mL and 86.12 mL, respectively. The mean VAS in men and women was 3.21 and 2.93, and 1.79 and 1.32 on the first and second postoperative days. There is no gender difference in the operating time, blood loss, VAS, TMJ symptoms, and lip numbness. The magnitude of mandibular setback was not correlated with immediate and long-term postoperative lip numbness. *Conclusion.* There are no gender differences in the intraoperative hemorrhage and postoperative sequelae (pain, lip numbness, and TMJ symptoms). In addition, neither symptom was significantly correlated with the amount of mandibular setback.

## 1. Introduction

Orthognathic surgery, which includes several procedures of varying complexity requiring high surgical skill, demands that surgeons consider multiple variables, such as the patient's overall physical condition, operating time, intraoperative hemorrhage, postoperative pain, and potential postoperative sequelae and complications. Not only are surgeons cautious about intraoperative hemorrhage and subsequent transfusion [1–4], patients are also concerned and may question the safety and risks of blood transfusion.

Postoperative pain and related sequelae are the most highly studied topics in modern medicine. Because of inappropriate postoperative analgesia, patients would reflect a negative emotion and decrease satisfaction on the postoperative quality of life. Orthognathic surgery involves relocation of the mandible, and potential postoperative complications must be realistically discussed by the surgeons and patients, such as whether the temporomandibular joint will exhibit clicking symptoms, whether mandibular mobility and mouth opening will recover, and whether abnormal lip sensation will occur.

For the correction of mandibular prognathism, several improvements in orthognathic surgery have emerged, with sagittal split ramus osteotomy (SSRO) and intraoral vertical ramus osteotomy (IVRO) being among the most popular of the current techniques. These two surgical methods differ in the operating time, hemorrhage volume, and the risk of postoperative sequelae and complications. While most studies focus on SSRO, very few reports discuss IVRO. Therefore, the present study aims to investigate the intraoperative hemorrhage and postoperative sequelae occurring in patients who undergo IVRO to treat mandibular prognathism.

## 2. Materials and Methods

A total of 80 patients with mandibular prognathism who were hospitalized at the Department of Dentistry and Maxillofacial Surgery and meeting the following criteria were enrolled: (1) absence of facial asymmetry, (2) absence of facial injury or other congenital facial deformities, (3) undergoing bilateral IVRO with no additional procedures, (4) 6-week maxillomandibular fixation period, and (5) at least 6 months of follow-up. The surgical duration and hemorrhage volume were recorded in all 80 patients, the change in the pre- and postoperative lip sensation was recorded in 69 patients (42 women and 27 men), the postoperative pain in Visual Analogue Scale (VAS) scores during hospitalization in 47 patients (28 women and 19 men), and the change between the pre- and postoperative maximum mouth opening (MMO) and temporomandibular joint (TMJ) clicking symptoms in 32 patients (22 women and 10 men).

All surgeries were performed under hypotensive anesthesia. The mean arterial pressure (MAP) was maintained at 60 mmHg to minimize hemorrhage. During hospitalization, an intravenous nonsteroidal anti-inflammatory drug (Aspegic, 0.5 g) was prescribed for pain control at 6-hour intervals. The operating time, volume of intraoperative hemorrhage, and the change between the pre- and postoperative blood components, pain in VAS (0–10 cm), postoperative lip sensation, TMJ clicking, and MMO were recorded during hospitalization. The correlation between the blood loss volume and postoperative VAS with the gender, age, operating time, and change in blood components was assessed using the *t*-test and Spearman rank correlation coefficient. The correlation between the magnitude of mandibular setback with the presence of TMJ clicking symptoms and lip sensation was also assessed by the Chi-square and Logistic regression tests.

## 3. Results

In analysis by sex, there was no significant difference among the patients in age, operative time, and blood loss volume (Table 1). The mean operating time and blood loss volume for men and women were 249.52 min and 229.39 min, and 104.03 mL and 86.12 mL, respectively, indicating the absence of any gender variation. The mean magnitude of mandibular setback and change in blood components was significantly greater in men than in women. The mean VAS in men and women was 3.21 and 2.93, and 1.79 and 1.32 on the first and second postoperative days, respectively, suggesting that the VAS did not differ significantly between genders (Table 2). The VAS was significantly lower on the second day than on the first day in both genders.

For patients exhibiting TMJ clicking preoperatively, the incidence in the right and left joints was 2/10 and 2/10 in men, and 5/22 and 4/22 in women, respectively. For patients exhibiting TMJ clicking postoperatively, the incidence in the right and left TMJs was 1/10 and 2/10 in men, and 1/22 and 3/22 in women, respectively. There is no gender difference in the TMJ symptoms (Table 2). The preoperative MMO did not differ between males and females, whereas postoperative MMO of males was significantly greater than females. Moreover, postoperative MMO of females was significantly smaller than their preoperative MMO.

The amount of left and right mandibular setback was 11.11 mm and 11.27 mm in men, and 9.01 mm and 9.60 mm in women, respectively (Table 1). The mandibular setback was significantly higher in men than in women. The incidence of patients reporting lower lip numbness immediately after surgery was 5/27 and 3/27 on the right and left sides in men, respectively, and 2/42 and 2/42 on the right and left sides in women, respectively (Table 2). At the final follow-up examination, only two male patients still experienced right-sided lower lip numbness, whereas all the female patients recovered sensation in both sides of the lower lip.

Spearman rank correlation coefficient analysis was used to assess the correlation between the blood loss volume and age, operating time, magnitude of mandibular setback, and change in blood components. Hemorrhage and operating time were positively correlated in men only (Table 3). The correlation analysis of the VAS on postoperative days 1 and 2 during hospitalization revealed that the decrease in postoperative Hct (%) and the day 2 VAS were positively correlated in women and negatively correlated in men (Table 4). When comparing the correlation between pre- and postoperative TMJ symptoms, our results showed significant reduction in the right side (Table 5). The magnitude of mandibular setback was not correlated with immediate and long-term postoperative lip numbness or abnormal sensation in male or female patients (Table 6).

## 4. Discussion

Orthognathic surgery is performed on the maxillofacial region, which is extensively vascularized, and certain intraoral maneuvers may have a limited field of vision. Thus, operative techniques must be highly accurate because hemostasis can be difficult to achieve. A study by Moenning et al. [1] revealed that the mean volume of hemorrhage was 176.6 mL in 171 bilateral SSRO patients, none of whom required transfusion. Similarly, our IVRO patients did not receive transfusion, and the mean volume of hemorrhage was 93 mL (women, 86.12 mL; men, 104.03 mL). Compared with SSRO [1], our IVRO patients had the advantage of a lower intraoperative hemorrhage. Ueki et al. [3] reported a significant positive correlation between intraoperative hemorrhage and operative time, whereas Böttger et al. [4] found that the correlation was low. In Table 3, our study revealed a difference between the genders, with the blood loss volume and operating time showing a positive correlation in men and no correlation in women. We also investigated the relationship between the blood loss volume and operating time with the mandibular setback amount and did not find any significant correlation in men or women. Therefore, we inferred that vascularity and perfusion were more abundant in men than in women, causing greater hemorrhage and a longer operating time to achieve hemostasis in male patients.

Many methods [5–7] are currently employed to minimize intraoperative hemorrhage and avoid the need for transfusion, among which hypotensive anesthesia is a well-established and effective technique that can reduce blood loss by 40% in orthognathic surgery in some reports. Hypotensive anesthesia can reduce blood flow, improve visibility in the operative field, and increase the efficacy of surgery and hemostasis, shortening the operating time. The volume of hemorrhage is also reduced, which further lowers the chance of requiring blood transfusion. As suggested by current data [8, 9], a MAP between 50 and 65 mmHg is safe in young patients because it does not interfere with perfusion to the brain, heart, kidney, and liver. However, close physiological monitoring during surgery and good communication between the surgeon and anesthesiologist is critical to ensure safety during hypotensive anesthesia.

Pain is a complex response involving central neuron-glial interactions during neuron transduction [10]. Postoperative pain following orthognathic surgery is not only caused by the operation itself, but also caused by surgical inflammatory reaction, surrounding muscle stiffness, and contraction of peripheral soft tissues. All of these events can induce the changes of pain perception in the central neuron system [10]. Our study revealed that postoperative pain did not differ in men and women, as both genders showed a significantly lower VAS on day 2 than on day 1 postoperatively. However, when the relationship between age and pain was evaluated, the data showed that older men had a greater reduction in VAS on day 2 postoperatively, probably because men may exhibit greater cognition and tolerance for pain as they age. Niederhagen et al. [11] reported that postoperative pain was highly correlated with operating time. However, our study found that postoperative pain had no significant correlation with operation time, intraoperative hemorrhage, and amount of mandibular setback either. Evans et al. [12] studied 45 patients who underwent orthognathic surgery and concluded that postoperative pain was generally not severe enough to justify administering strong opioid analgesics. Postoperative administration of nonsteroidal anti-inflammatory drugs (NSAIDs) has been widely demonstrated to effectively alleviate pain and reduce morphine dosage. We also found that NSAIDs were sufficient to control postoperative pain. We evaluated the patients' postoperative VAS scores and found them comparable to the preoperative orthodontic pain in VAS scores. This finding can greatly facilitate preoperative communication between surgeons and patients and allow surgeons to inform patients about postoperative pain, reducing the anxiety and pressure associated with surgery.

The method of orthognathic surgery can also affect the recovery of mandibular mobility and the time required for recovery. Some studies [13, 14] show that intraoperative internal fixation of the mandibles or postoperative intermaxillary fixation may affect future mandibular mobility. SSRO employs internal fixation but not intermaxillary fixation; therefore, mandibular mobility can be resumed immediately postoperatively. By contrast, IVRO uses intermaxillary fixation but not internal fixation; therefore, the mandible is immobilized for 6 weeks postoperatively. MMO measurement is a simple and easy way to assess the postoperative recovery of mandibular mobility. Boyd et al. [13] reported that SSRO and IVRO do not differ in the MMO recovery. The MMO has been significantly correlated with intermaxillary fixation during the 6 months after surgery, a correlation that disappears 1 year postoperatively. The preoperative MMO did not differ between our male and female patients, but women had a significantly lower MMO than men at the final follow-up examination. There was no difference in the preoperative and final MMO in the male patients. The final MMO in female patients was significantly lower than the preoperative MMO by approximately 3 mm. We suspect that the male patients may have paid greater attention during the mouth opening exercise, while the female patients may have considered it acceptable to exceed the MMO target of 45 mm because normal speech and eating do not require reaching MMO.

How orthognathic surgery affects TMD is still under debate [15–17]; some scholars believe it can alleviate TMD symptoms, whereas others report that orthognathic surgery may induce or worsen TMD symptoms. TMJ clicking is the most common TMD symptom in patients. There was no gender variation in the patients exhibiting TMJ clicking before surgery and at the final examination. However, after surgery, women had significantly decreased TMJ clicking on the right side. The number of women with right-sided TMJ clicking decreased from five people to one person. The four women with left-sided TMJ clicking initially improved with no symptoms postoperatively, while an additional three women developed symptoms after surgery. The number of men with right-sided TMJ clicking decreased from two to one, and two men with left-sided TMJ clicking remained symptomatic after surgery. Based on these findings, it remains unclear whether orthognathic surgery improves or induces TMJ clicking symptoms. We also found that the amount of mandibular setback was not correlated with postoperative TMJ clicking in either gender.

Some researchers [18–20] reported that SSRO increased the risk of neurosensory damage in the lower lip than IVRO. The currently accepted reasons for this difference include peeling of the inner periosteum of the ramus, exposure of the inferior alveolar nerve during SSRO splitting, crushing of the nerves during bone segment fixation, and postoperative edema. The distal segment may press against the nerves during backward replacement. Rigid (screw) and nonrigid (wire) fixation methods can be used in SSRO to fix the two bone segments. Lemke et al. [21] reported that rigid fixation caused greater mental nerve numbness than wire fixation in SSRO patients examined by brush stroke determination. This finding indicates that rigid fixation compresses the nerves more strongly than wire fixation. By contrast, because IVRO does not split the bones or fix the bone segments, there is a lower risk of injuring the mandibular canal or compressing the inferior alveolar nerve and less extension of the vascular/nerve bundles caused by distal segment movement. Among our patients, approximately 9% (12/138) showed lower lip numbness postoperatively, and only 2% still reported numbness at the final follow-up, with reduced symptoms. In addition, there was no gender difference in the incidence of lower lip numbness immediately after surgery or at the final follow-up. When investigating whether the amount of mandibular setback increased the incidence of postoperative lower lip numbness, we did not find any correlation, indicating that accurately determining the bone cutting position and carefully overlapping the two bone segments can reduce extension of the inferior alveolar nerve bundle and decrease the occurrence of lower lip numbness.

## 5. Conclusion

Even with a larger amount of mandibular setback, our patients presented a lower intraoperative hemorrhage without blood transfusion. There are no gender differences in the intraoperative hemorrhage and postoperative sequelae (pain, lip numbness, and TMJ symptoms). In addition, neither symptom was significantly correlated with the amount of mandibular setback. The 6-month postoperative MMO of males and females were returned to normal range. Therefore, IVRO is a reliable technique without intraoperative and postoperative sequelae.

## Figures and Tables

**Table 1:** Mean differences in patients' characteristics by sex (*n* = 80).

	Female	Male	
Parameters	(*n* = 49)	(*n* = 31)	*P*
	Mean (SD)	Mean (SD)	
Age, y	23.31 (4.06)	22.42 (3.73)	0.327
Setback, mm			
Right side	9.60 (3.31)	11.27 (3.41)	0.037^*∗*^
Left side	9.01 (3.34)	11.11 (3.57)	0.012^*∗*^
Operation time, min	229.39 (40.82)	249.52 (48.86)	0.065
Blood loss, mL	86.12 (54.98)	104.03 (56.73)	0.175
Preoperation			
RBCs, ×10^6^/*μ*L	4.51 (0.45)	5.10 (0.60)	<0.001^†^
Hgb, g/dL	13.21 (1.06)	15.13 (1.00)	<0.001^†^
Hct, %	39.35 (2.80)	44.90 (2.83)	<0.001^†^
Postoperation			
RBCs, ×10^6^/*μ*L	3.92 (0.37)	4.40 (0.55)	<0.001^†^
Hgb, g/dL	11.60 (1.15)	13.17 (0.94)	<0.001^†^
Hct, %	34.42 (3.01)	38.73 (2.75)	<0.001^†^
Reduced blood ingredients			
RBCs, ×10^6^/*μ*L	0.59 (0.23)	0.70 (0.22)	0.034^*∗*^
Hgb, g/dL	1.61 (0.61)	1.96 (0.74)	0.035^*∗*^
Hct, %	4.93 (1.75)	6.17 (1.98)	0.007^*∗*^

*n* = number of patients.

*t*-test statistics; *P* values for differences by sex.

^*∗*^ *P* < 0.05; ^†^ *P* < 0.001.

**Table 2:** Mean differences in postoperation symptoms by sex.

Variables	Female (F) Mean (SD)	Male (M) Mean (SD)	*P*
VAS, cm; F/M = 28/19			
D1	2.93 (1.03)	3.21 (1.36)	0.460
D2	1.32^a^ (1.49)	1.79^a^ (1.32)	0.275
TMJ clicking, *n*; F/M = 22/10			
Preoperation			
Right side	0.23 (0.42)	0.20 (0.40)	0.868
Left side	0.18 (0.39)	0.20 (0.40)	0.910
Postoperation			
Right side	0.05^b^ (0.21)	0.10 (0.30)	0.628
Left side	0.14 (0.34)	0.20 (0.40)	0.683
MMO, mm; F/M = 22/10			
Preoperation	54.66 (6.28)	57.25 (8.73)	0.435
Immediate after removal of IMF	19.27 (5.48)	22.20 (3.99)	0.116
2 weeks after removal of IMF	40.84 (6.86)	42.09 (9.34)	0.723
Final observation	51.05^c^ (4.67)	57.90 (6.41)	0.012^*∗*^
Lip numbness, *n*; F/M = 42/27			
Right side			
Immediate after operation	0.05 (0.21)	0.19 (0.39)	0.107
Final examination	0.00 (0.00)	0.07 (0.26)	0.161
Left side			
Immediate after operation	0.05 (0.21)	0.11 (0.31)	0.370
Final examination	0.00 (0.00)	0.00 (0.00)	1.000

*n* = number of sides; MMO = maximum mouth opening.

*t*-test statistics; *P* values for differences by sex.

^*∗*^ *P* < 0.05.

D1: postoperation day 1; D2: postoperation day 2.

^a^Significant difference between D1 and D2.

^b^Significant difference between preoperation and postoperation.

^c^Significant difference between preoperation and final examination.

**Table 3:** Correlation between blood loss and parameters in Spearman rank correlation analysis.

	Correlation coefficient	
Parameters	Female	Male
	(*n* = 49)	(*n* = 31)
Age, y	0.138	0.226
Operation time, min	−0.008	0.396^*∗*^
Reduced blood ingredients		
RBCs, ×10^6^/*μ*L	0.021	0.060
Hgb, g/dL	0.056	0.034
Hct, %	0.067	0.036
Setback, mm		
Right side	0.221	0.021
Left side	−0.091	−0.110

Spearman rank correlation coefficients: ^*∗*^ *P* < 0.05.

**Table 4:** Correlation between VAS and parameters in Spearman rank correlation analysis.

Parameters	Correlation coefficient			
	Female		Male	
	(*n* = 28)		(*n* = 19)	
	D1	D2	D1	D2
Age, y	0.197	−0.051	−0.256	−0.485^*∗*^
Operation time, min	0.264	0.174	−0.027	0.076
Blood loss, mL	−0.293	0.074	0.061	−0.222
Reduced blood ingredients				
RBCs, ×10^6^/*μ*L	−0.083	0.298	0.115	0.01
Hgb, g/dL	−0.231	0.150	0.177	0.029
Hct, %	−0.065	0.409^*∗*^	0.190	0.058
Setback, mm				
Right side	−0.240	−0.150	−0.014	−0.086
Left side	−0.111	0.092	0.033	−0.207

Spearman rank correlation coefficients: ^*∗*^ *P* < 0.05.

D1: postoperation day 1.

D2: postoperation day 2.

**Table 5:** The TMJ symptoms changed before and after orthognathic surgery in the Chi-square and logistic regression tests.

	Postoperation TMJ clicking	
Parameters	Right (*n* = 32)	Left (*n* = 32)
	*P* value	*P* value
Preoperation clicking^*∗*^		
Right/left	0.006	0.185
Setback, mm^*∗∗*^		
Right/left, ≦9 mm	—	0.658
Right/left, >9 mm	0.758	0.948

^*∗*^Chi-square test; significant *P* < 0.05.

^*∗∗*^Logistic regression test; significant *P* < 0.05.

—: nonestimated (due to no TMJ clicking).

**Table 6:** The lower lip numbness changed at the immediate and final examination in the logistic regression test.

	Postoperation lower lip numbness	
Parameters	Right (*n* = 69)	Left (*n* = 69)
	*P* value	*P* value
Setback, ≦9 mm		
Immediate after operation	0.481	0.325
Final examination	0.961	—
Setback, >9 mm		
Immediate after operation	0.812	0.432
Final examination	0.378	—

Logistic regression test; significant *P* < 0.05.

—: nonestimated (due to no lip numbness).

## References

[1] Moenning J. E., Bussard D. A., Lapp T. H., Garrison B. T. (1995). Average blood loss and the risk of requiring perioperative blood transfusion in 506 orthognathic surgical procedures. *Journal of Oral and Maxillofacial Surgery*.

[2] Chen H.-S., Lai S. S.-T., Lee K.-T., Lee H.-E., Hsu K.-J. (2014). Intraoperative blood loss during an osteotomy of the bilateral vertical ramus. *Journal of Dental Sciences*.

[3] Ueki K., Marukawa K., Shimada M., Nakagawa K., Yamamoto E. (2005). The assessment of blood loss in orthognathic surgery for prognathia. *Journal of Oral and Maxillofacial Surgery*.

[4] Böttger S., Streckbein P., Hartmann B., Schaaf H., Howaldt H. P., Junger A. (2009). Retrospective analysis of autologous blood use in bimaxillary repositioning osteotomy surgery: a quality improvement study. *Transfusion*.

[5] Praveen K., Narayanan V., Muthusekhar M. R., Baig M. F. (2001). Hypotensive anaesthesia and blood loss in orthognathic surgery: a clinical study. *British Journal of Oral and Maxillofacial Surgery*.

[6] Yu C. N. F., Chow T. K., Kwan A. S. K., Wong S. L., Fung S. C. (2000). Intra-operative blood loss and operating time in orthognathic surgery using induced hypotensive general anaesthesia: prospective study. *Hong Kong Medical Journal*.

[7] Chen C.-M., Lai S. S.-T., Hsu K.-J., Lee H.-E., Huang H.-L. (2011). Assessment of the related factors of blood loss and blood ingredients among patients under hypotensive anesthesia in orthognathic surgery. *Journal of Craniofacial Surgery*.

[8] Rodrigo C. (1995). Induced hypotension during anesthesia with special reference to orthognathic surgery. *Anesthesia Progress*.

[9] Kurian A., Ward-Booth P. (2004). Blood transfusion and orthognathic surgery—a thong of the past?. *British Journal of Oral and Maxillofacial Surgery*.

[10] Arthur C., Guyton M. D., Hall J. E. (2000). *Somatic Sensation II. Textbook of Medical Physiology*.

[11] Niederhagen B., Braumann B., Dierke-Dzierzon C., Albrecht S. (1997). Postoperative pain after interventions in the area of the mouth-jaw-face. *Mund-, Kiefer- und Gesichtschirurgie*.

[12] Evans C. C., Levine B., Bahn S. (1976). Analgesic requirements after orthognathic surgery. *Journal of Oral Surgery*.

[13] Boyd S. B., Karas N. D., Sinn D. P. (1991). Recovery of mandibular mobility following orthognathic surgery. *Journal of Oral and Maxillofacial Surgery*.

[14] Al-Belasy F. A., Tozoglu S., Dolwick M. F. (2013). Mandibular hypomobility after orthognathic surgery: a review article. *Journal of Oral and Maxillofacial Surgery*.

[15] Farella M., Michelotti A., Bocchino T., Cimino R., Laino A., Steenks M. H. (2007). Effects of orthognathic surgery for class III malocclusion on signs and symptoms of temporomandibular disorders and on pressure pain thresholds of the jaw muscles. *International Journal of Oral and Maxillofacial Surgery*.

[16] Kalha A. (2010). Orthognathic treatment and temporomandibular disorders part 2. *Evidence-Based Dentistry*.

[17] Al-Riyami S., Cunningham S. J., Moles D. R. (2009). Orthognathic treatment and temporomandibular disorders: a systematic review. Part 2. Signs and symptoms and meta-analyses. *American Journal of Orthodontics & Dentofacial Orthopedics*.

[18] Alolayan A. B., Leung Y. Y. (2014). Risk factors of neurosensory disturbance following orthognathic surgery. *PLoS ONE*.

[19] Takazakura D., Ueki K., Nakagawa K. (2007). A comparison of postoperative hypoesthesia between two types of sagittal split ramus osteotomy and intraoral vertical ramus osteotomy, using the trigeminal somatosensory-evoked potential method. *International Journal of Oral and Maxillofacial Surgery*.

[20] Hashiba Y., Ueki K., Marukawa K. (2007). A comparison of lower lip hypoesthesia measured by trigeminal somatosensory-evoked potential between different types of mandibular osteotomies and fixation. *Oral Surgery, Oral Medicine, Oral Pathology, Oral Radiology and Endodontology*.

[21] Lemke R. R., Rugh J. D., Van Sickels J., Bays R. A., Clark G. M. (2000). Neurosensory differences after wire and rigid fixation in patients with mandibular advancement. *Journal of Oral and Maxillofacial Surgery*.

